# Comparative genomic analysis of *Acinetobacter baumannii* clinical isolates reveals extensive genomic variation and diverse antibiotic resistance determinants

**DOI:** 10.1186/1471-2164-15-1163

**Published:** 2014-12-22

**Authors:** Fei Liu, Yuying Zhu, Yong Yi, Na Lu, Baoli Zhu, Yongfei Hu

**Affiliations:** CAS key Laboratory of Pathogenic Microbiology and Immunology, Institute of Microbiology, Chinese Academy of Sciences, Beijing, 100101 China; Beijing Key Laboratory of Microbial Drug Resistance and Resistome, Beijing, 100101 China; Collaborative Innovation Center for Diagnosis and Treatment of Infectious Diseases, The First Affiliated Hospital, College of Medicine, Zhejiang University, Hangzhou, 310006 China; The 306th Hospital of People’s Liberation Army, Beijing, 100101 China

**Keywords:** *Acinetobacter baumannii*, Multidrug resistance, Resistance island, SNP, Whole-genome sequencing

## Abstract

**Background:**

*Acinetobacter baumannii* is an important nosocomial pathogen that poses a serious health threat to immune-compromised patients. Due to its rapid ability to develop multidrug resistance (MDR), *A. baumannii* has increasingly become a focus of attention worldwide. To better understand the genetic variation and antibiotic resistance mechanisms of this bacterium at the genomic level, we reported high-quality draft genome sequences of 8 clinical isolates with various sequence types and drug susceptibility profiles.

**Results:**

We sequenced 7 MDR and 1 drug-sensitive clinical *A. baumannii* isolates and performed comparative genomic analysis of these draft genomes with 16 *A. baumannii* complete genomes from GenBank. We found a high degree of variation in *A. baumannii*, including single nucleotide polymorphisms (SNPs) and large DNA fragment variations in the AbaR-like resistance island (RI) regions, the prophage and the type VI secretion system (T6SS). In addition, we found several new AbaR-like RI regions with highly variable structures in our MDR strains. Interestingly, we found a novel genomic island (designated as GI_BJ4_) in the drug-sensitive strain BJ4 carrying metal resistance genes instead of antibiotic resistance genes inserted into the position where AbaR-like RIs commonly reside in other *A. baumannii* strains. Furthermore, we showed that diverse antibiotic resistance determinants are present outside the RIs in *A. baumannii*, including antibiotic resistance-gene bearing integrons, the *bla*_*OXA-23*_-containing transposon Tn*2009*, and chromosomal intrinsic antibiotic resistance genes.

**Conclusions:**

Our comparative genomic analysis revealed that extensive genomic variation exists in the *A. baumannii* genome. Transposons, genomic islands and point mutations are the main contributors to the plasticity of the *A. baumannii* genome and play critical roles in facilitating the development of antibiotic resistance in the clinical isolates.

**Electronic supplementary material:**

The online version of this article (doi:10.1186/1471-2164-15-1163) contains supplementary material, which is available to authorized users.

## Background

*A. baumannii*, an important nosocomial pathogen, is becoming an increasing threat to hospital patients due to its ability to develop multidrug resistance (MDR) [1–3]. Drug resistance in *A. baumannii* is due to a combination of mechanisms, including the expression of β-lactamases, alteration of cell membrane impermeability, and increased expression of efflux pumps [4]. The drug resistance genes of *A. baumannii* isolates are often clustered into antibiotic resistance islands (AbaRs) that interrupt the ATPase gene (*comM*) [5]. For example, a 86-kb resistance island (RI) was found in *A. baumannii* strain AYE (AbaR1) [6], and a shorter RI was identified in the *A. baumannii* strain ACICU (AbaR2) [7]. RIs are thought to emerge from the integration of plasmids or other mobile elements, and some drug-susceptible strains lack these RIs [6]. In addition, plasmid-borne resistance genes have also been reported, e.g., the *bla*_*OXA-23*_ gene, which is associated with carbapenem resistance, has been identified in clinical *A. baumannii* isolates around the world [8, 9].

Compared with current knowledge regarding antibiotic resistance mechanisms in *A. baumannii*, less is known regarding the virulence factors in this bacterium [10]. Several studies have focused on characterizing the formation of biofilms, one of the determinants involved in the pathogenesis in *A. baumannii*. For example, a chaperone-usher pili assembly system (csu locus) has been shown to be involved in attachment and biofilm formation in *A. baumannii* [11]. Other virulence factors identified include a siderophore-mediated iron acquisition system, AbaI autoinducer synthase, the BfmRS two-component regulatory system, the type VI secretion system (T6SS) [12] and lipopolysaccharide (LPS) [13]. The LPS found in *A. baumannii*, which is composed of lipids, O-antigen, and an outer core (OC) and inner core, has been shown to be a major contributor to the pathogenesis of infection [13]. The OC gene locus contains many genes encoding glycosyltransferase enzymes that catalyze the bonds between sugars in the OC structure [14].

Whole-genome sequencing studies comparing distinct drug-susceptible and MDR strains [1, 15] or isolates from a single patient [16] have improved our understanding of the evolution of *A. baumannii*. To better understand the genomic variation and the antibiotic resistance mechanisms in *A. baumannii*, here we sequenced eight clinical *A. baumannii* isolates with various sequence types and drug susceptibility profiles and performed comparative genomic analysis.

## Results

### Susceptibility profiles, multilocus sequence typing (MLST) and whole-genome sequencing

The susceptibility profiles for all sequenced strains are shown in Table 1. All 7 MDR strains were resistant to the antibiotics gentamicin (CN), ciprofloxacin (CIP), ceftriaxone (CTR), ceftazidime (CAZ), cefepime (FEP), and tetracycline (TE) but susceptible to polymyxin B (PB). The drug-sensitive strain BJ4 was sensitive or intermediate to all tested antibiotics except CTR.

**Table 1:** **Antimicrobial susceptibility profiles.R, resistant; I, intermediate; S, susceptible**

Antimicrobial classes	Antimicrobial drugs	MIC (mg/L) and Susceptibility							
		BJ1	BJ2	BJ3	BJ4	BJ5	BJ6	BJ7	BJ8
Aminoglycosides	Gentamicin (CN)	>8, R	>8, R	>8, R	≤1, S	>8, R	>8, R	>8, R	>8, R
	Amikacin (AK)	≤16, S	>32, R	>32, R	≤2, S	>32, R	≤16, S	≤16, S	>32, R
Antipseudomonal carbapenems	Imipenem (IPM)	>8, R	>8, R	>8, R	≤1, S	>8, R	>8, R	>8, R	≤1, S
Antipseudomonal fluoroquinolones	Ciprofloxacin (CIP)	>2, R	>2, R	>2, R	≤0.25, S	>2, R	>2, R	>2, R	>2, R
	Lavo-ofloxacin (LEV)	>4, R	>4, R	>4, R	≤0.25, S	>4, R	>4, R	>4, R	=4, I
Antipseudomonal penicillins and β-lactamase inhibitors	Piperacillin-tazobactam (TZP)	>64/4, R	>64/4, R	>64/4, R	≤4, I	>64/4, R	>64/4, R	>64/4, R	≤16/4, S
Extended-spectrum cephalosporins	Ceftriaxone (CTR)	>32, R	>32, R	>32, R	8, R	>32, R	>32, R	>32, R	>32, R
	Ceftazidime (CAZ)	>16, R	>16, R	>16, R	4, I	>16, R	>16, R	>16, R	>16, R
	Cefepime (FEP)	>16, R	>16, R	>16, R	2, S	>16, R	>16, R	>16, R	>16, R
Polymyxins	Polymyxin B (PB)	≤2, S	≤2, S	≤2, S	≤2, S	≤2, S	≤2, S	≤2, S	≤2, S
Tetracyclines	Tetracycline (TE)	=8, I	>8, R	>8, R	≤4, S	>8, R	>8, R	>8, R	>8, R

We found that all 7 MDR strains correspond to global clone II (GC II). The strains BJ2, BJ6, and BJ7 share the same sequence type (ST), namely, ST208, and strains BJ1 and BJ5 share a type (ST191). In addition, strains BJ3 and BJ8 belong to ST218 and ST368, respectively. However, the drug-sensitive strain BJ4 shows a novel sequence type.

The basic whole-genome sequencing statistics are shown in Table 2. Illumina 100 bp paired-end sequencing produced more than 900 Mb of data for each of the eight strains, and the sequencing depth ranged from 239× to 473×. The GC content of the genomes was approximately 38.9%, as expected for the species. The size of the genomes varied from 3.86 to 4.03 Mb.

**Table 2:** **Sequencing statistics for the** ***A. baumannii*** **isolates**

Strain	Raw data (Mb)	Sequencing depth (X)	Scaffold number	N50 length	Mean length	Max length	Full length (bp)	GC
BJ1	979	246	44	203665	90399	458403	3977574	38.9%
BJ2	959	239	52	164054	77219	364231	4015419	38.9%
BJ3	1540	390	35	236595	112903	460149	3951629	38.9%
BJ4	1666	420	54	150346	73375	433944	3962297	38.9%
BJ5	1895	473	47	211748	85260	458351	4007238	38.9%
BJ6	1079	268	56	133586	71990	364278	4031457	38.9%
BJ7	961	239	54	133586	74623	364278	4029682	38.9%
BJ8	1193	309	56	170785	68971	466960	3862420	39.0%

### Phylogenetic analysis of *A. baumannii*isolates

**Figure 1:**
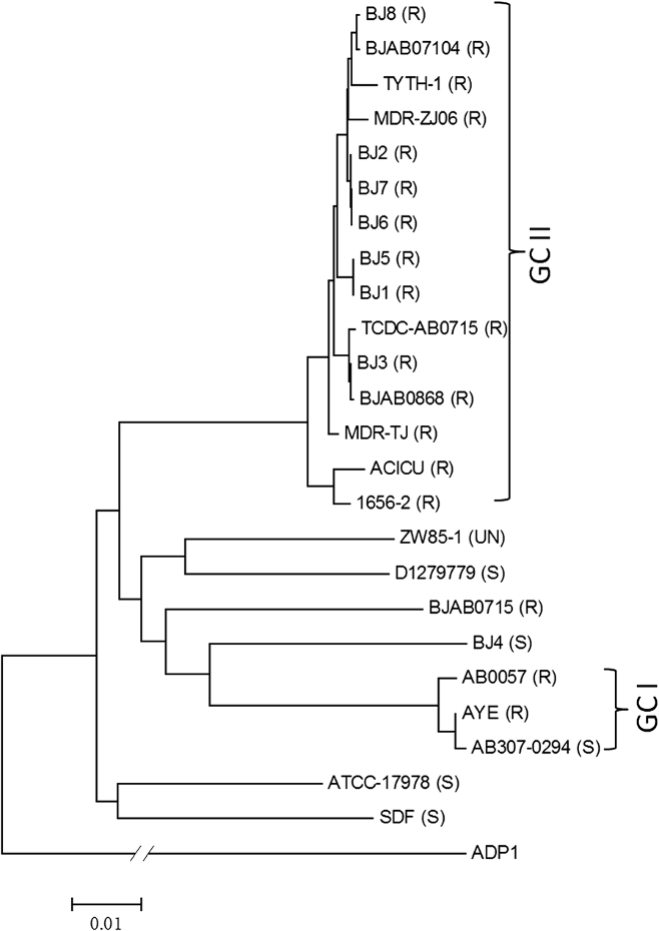
**Phylogenetic tree of** ***A. baumannii*** **isolates.** A maximum likelihood tree was constructed using dnaml from the PHYLIP package, based on the core SNP in each genome. Bar, 0.01 substitution per nucleotide. “R” = multidrug resistance; “S” = drug-sensitive; “UN” = unknown.

A maximum-likelihood tree of the 8 sequenced genomes and 16 reported *A. baumannii* complete genomes were created based on core SNPs from whole-genome alignment (Figure 1). The phylogenetic tree showed that the previously sequenced strains and all of the 7 MDR clinical isolates belonging to GC II formed a clade, while strains AB307-0294, AYE, and AB0057, which belong to GC I, grouped together. The BJ1 and BJ5 strains are closely related, while strains BJ2, BJ6, and BJ7 form another closely related group. Interestingly, strain BJ4, the drug-sensitive strain, is distinct from all of the sequenced MDR strains, which may indicate that it has a unique origin compared with other drug-resistant strains.

### *A. baumannii*core and unique genes

**Figure 2:**
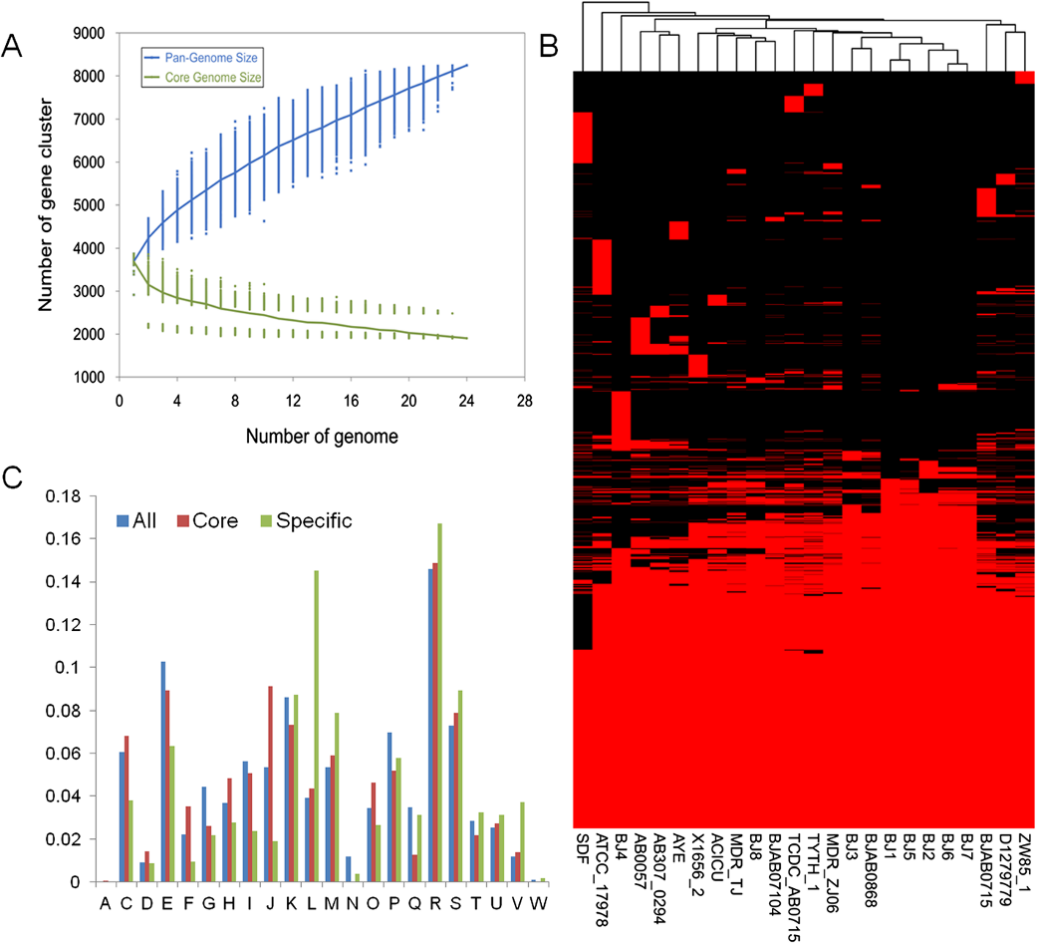
**Analysis of the core and pan-genome of** ***A. baumannii*** **isolates. (A)** Core and pan-genomic calculations in *A. baumannii* isolates. Each green point represents the number of genes conserved between genomes. All of the points are plotted as a function of the strain number (x). The deduced pan-genome size: P(x) = 972×^0.54^ + 2786.22. The height of the curve continues to increase because the pan-genome of *A. baumannii* is open. **(B)**. Genes missing or present in *A. baumannii* isolates. The heat map illustrates the distribution of core and accessory genes across the *A. baumannii* strains. The columns represent *A. baumannii* isolates. The rows represent genes. The red and black regions represent the presence or absence of genes in a particular genome, respectively. The black regions indicate features missing in that strain but present in one or more of the other *A. baumannii* strains. **(C)**. The distribution of all, core, and specific genes according to the COG classification. The *y*-axis indicates the percentage of genes in various COG categories. (U) Intracellular trafficking and secretion; (V) Defense mechanisms; (D) Cell cycle control, mitosis, and meiosis; (F) Nucleotide transport and metabolism; (O) Post-translational modification, protein turnover, chaperones; (O) Posttranslational modification, protein turnover, chaperones; (J) Translation, ribosomal structure and biogenesis; (H) Coenzyme transport and metabolism; (M) Cell wall/membrane/envelope biogenesis; (S) Function unknown; (K) Transcription; (P) Inorganic ion transport and metabolism; (T) Signal transduction mechanisms; (G) Carbohydrate transport and metabolism; (N) Cell motility; (C) Energy production and conversion; (L) Replication, recombination, and repair; (E) Amino acid transport and metabolism; (I) Lipid transport and metabolism; (R) General function prediction only; (Q) Secondary metabolites biosynthesis, transport, and catabolism.

We compared the gene contents of the 8 genomes with other *A. baumannii* reference genomes using the PanOCT analysis software [17], which utilizes conserved gene neighborhood (CGN) and frameshift detection in a weighted scoring scheme and the BLAST score ratio to effectively generate non-paralogous gene clusters. We found that the pan-genome continued to expand after the compilation of 24 genomes, whereas the number of core genes remained relatively stable with the addition of new strains (Figure 2A). The size of the pan-genome was 8245 genes, and there are 1902 genes (core) shared among the 24 isolates (Figure 2B). The number of unique genes ranges from 7 in strain BJ1 to 552 in strain SDF (Table 3). Many of these unique genes are hypothetical, transposon-related and phage-related genes. Detailed information regarding orthologous groups and singletons of the strains is provided in Additional file 1: Table S1. The large number of unique genes in these genomes likely indicates frequent horizontal gene transfer events in *A. baumannii*. Hierarchical clustering of these strains based on gene content yields a dendrogram (Figure 2B) that is similar to the core SNP-based phylogenetic tree (Figure 1) in which strains from GC I form one group and strains from GC II form another group.We further analyzed the core and unique genes according to the various classes of the Clusters of Orthologous Groups (COGs) (Figure 2C). We found that core genes were significantly enriched in genes belonging to class J (Translation, ribosomal structure and biogenesis; P value = 1.53e-09) and class F (Nucleotide transport and metabolism; P value = 0.0008301). In contrast, unique genes were significantly enriched in class L (Replication, recombination and repair; P value < 2.2e-16), class V (Defense mechanisms; P value = 1.868e-09), and class M (Cell wall/membrane/envelope biogenesis; P value = 0.0005701).

**Table 3:** **Orthologous clusters in the** ***A. baumannii*** **pan-genome**

Strain	Total	Non-core	% Non-core	Unique	% Unique
BJ1	3808	1906	50.1%	7	0.2%
BJ2	3846	1944	50.5%	32	0.8%
BJ3	3747	1845	49.2%	10	0.3%
BJ4	3767	1865	49.5%	408	10.8%
BJ5	3833	1931	50.4%	9	0.2%
BJ6	3870	1968	50.9%	8	0.2%
BJ7	3869	1967	50.8%	9	0.2%
BJ8	3651	1749	47.9%	27	0.7%
1656_2	3715	1813	48.8%	159	4.3%
AB0057	3790	1888	49.8%	182	4.8%
AB307_0294	3458	1556	45.0%	76	2.2%
ACICU	3667	1765	48.1%	70	1.9%
ATCC_17978	3787	1885	49.8%	507	13.4%
AYE	3607	1705	47.3%	183	5.1%
BJAB07104	3755	1853	49.3%	32	0.9%
BJAB0715	3848	1946	50.6%	247	6.4%
BJAB0868	3703	1801	48.6%	35	0.9%
D1279779	3388	1486	43.9%	105	3.1%
MDR_TJ	3704	1802	48.7%	28	0.8%
MDR_ZJ06	3860	1958	50.7%	58	1.5%
SDF	2913	1011	34.7%	552	18.9%
TCDC_AB0715	3851	1949	50.6%	178	4.6%
TYTH_1	3680	1778	48.3%	130	3.5%
ZW85_1	3465	1563	45.1%	136	3.9%

To compare protein sequence evolution rates between the MDR isolates and the drug-sensitive isolates, we measured the nonsynonymous substitution rate (Ka or dN) in 1902 orthologous genes. We previously showed that this rate is a relatively consistent parameter for defining fast-evolving and slow-evolving protein-coding genes [18]. The fast-evolving genes we identified among the MDR isolates include many outer membrane proteins and stress-related proteins; one of these proteins is a phenazine biosynthetic PhzF-like protein that serves as an enzyme essential for phenazine synthesis. Phenazines are pigments, and many exhibit broad-spectrum antibiotic activity against bacteria, fungi, and parasites and can contribute to the ecological competence of the strains [19]. In contrast, the identified slow-evolving genes include many conserved hypothetical proteins and metabolism-related proteins. For example, SbmA, which is involved in the prokaryotic internalization of antimicrobial peptides (AMPs), was identified as a slow-evolving gene [20].

### SNPs among *A. baumannii*strains

The number of SNPs among the 7 MDR strains with distinct STs ranged from 920 to 2675 (Additional file 2: Table S2). The strains with the same STs showed fewer SNPs, ranging from 74 to 196. Among the 74 putative SNPs identified between BJ6 and BJ7, only 12 (16%) were nonsynonymous mutations; these SNPs were located within genes coding for outer membrane receptor for monomeric catechols, dihydropteroate synthase, fatty acid desaturase, and a putative RND superfamily exporter. We also found similar nonsynonymous mutations within all of the genes mentioned above between BJ1 and BJ5.

To identify SNP regions clustered among the 7 MDR strains, SNP density was estimated throughout the genomes using a sliding window of 5 kb. The resulting SNP density map shows a non-random distribution, with many regions having elevated SNP density (Additional file 3: Figure S1). One large region of elevated SNP density is around the origin of replication of the genome and the K locus, as reported by Snitkin et al. [21]. We also found other SNP clusters containing genes involved in heme utilization, arginine and proline metabolism, the ABC-type transport system, etc.

### Virulence genes identified in the Virulence Factors Database (VFDB)

Putative virulence genes were identified by aligning ORF protein sequences to the virulence factors in the VFDB (Additional file 4: Table S3). The homologs of *clpP* (ATP-dependent Clp protease proteolytic subunit), *aldA* (aldehyde dehydrogenase), *xcpR* (general secretion pathway protein E), *ureA* (urease alpha subunit), *tviB* (Vi polysaccharide biosynthesis protein), *pilG* (twitching motility protein), *pilH* (twitching motility protein), *htpB* (60 K heat shock protein), *sodB* (superoxide dismutase) and *manB* (phosphomannomutase) were present in all of the *A. baumannii* strains. The homologs of *pilC*, *pilT*, and *pilU* were absent in SDF but present in the other strains. In addition, the homologs of *bplB* (putative acetyltransferase), *VC0817* (putative transposase), *SF2983* (transposase of Tn10) and *katB* (catalase-peroxidase) were exclusively present in ATCC 17978, BJ4, AYE and D1279779, respectively.

### Large genomic variants among *A. baumannii*strains

**Figure 3:**
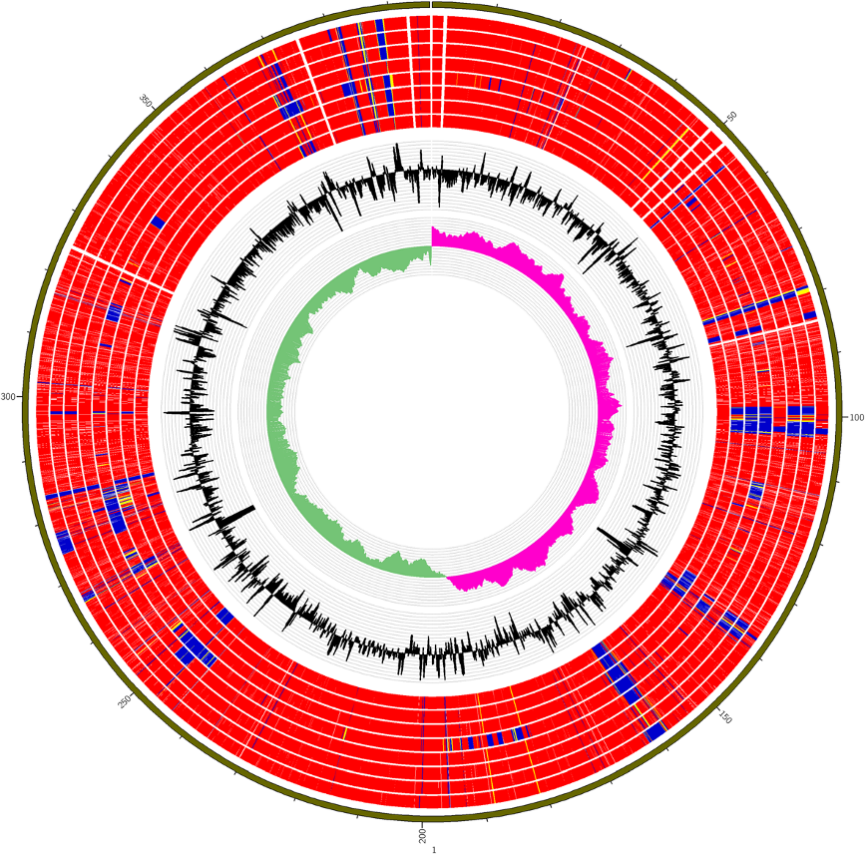
**ORF comparisons in** ***A. baumannii*** **genomes.** Proteins from all sequenced 8 *A. baumannii* strains were aligned using MDR-TJ as a reference. The tracks from inside to outside indicate the GC screw and GC content, and the circles from inside to outside are the BLASTP percent identities of ORFs from BJ1 to BJ8 against MDR-TJ. Red indicates 90–100% identity, yellow indicates 60–89% identity, and blue indicates 0–59% identity.

We compared the genomes of our 8 *A. baumannii* strains with the reference genome of *A. baumannii* MDR-TJ, a multidrug resistance strain belonging to GC II group [22]. We identified many highly variable regions (Figure 3); specifically, the following regions on the MDR-TJ genome are missing or have low identity with our strains: from 982 to 1,034 kb, 1,343 to 1,363 kb, 1,364 to 1,400 kb, 1,575 to 1,617 kb, 2,460 to 2,500 kb, 3,672 to 3,710 kb and 3,798 to 3,894 kb.

**Figure 4:**
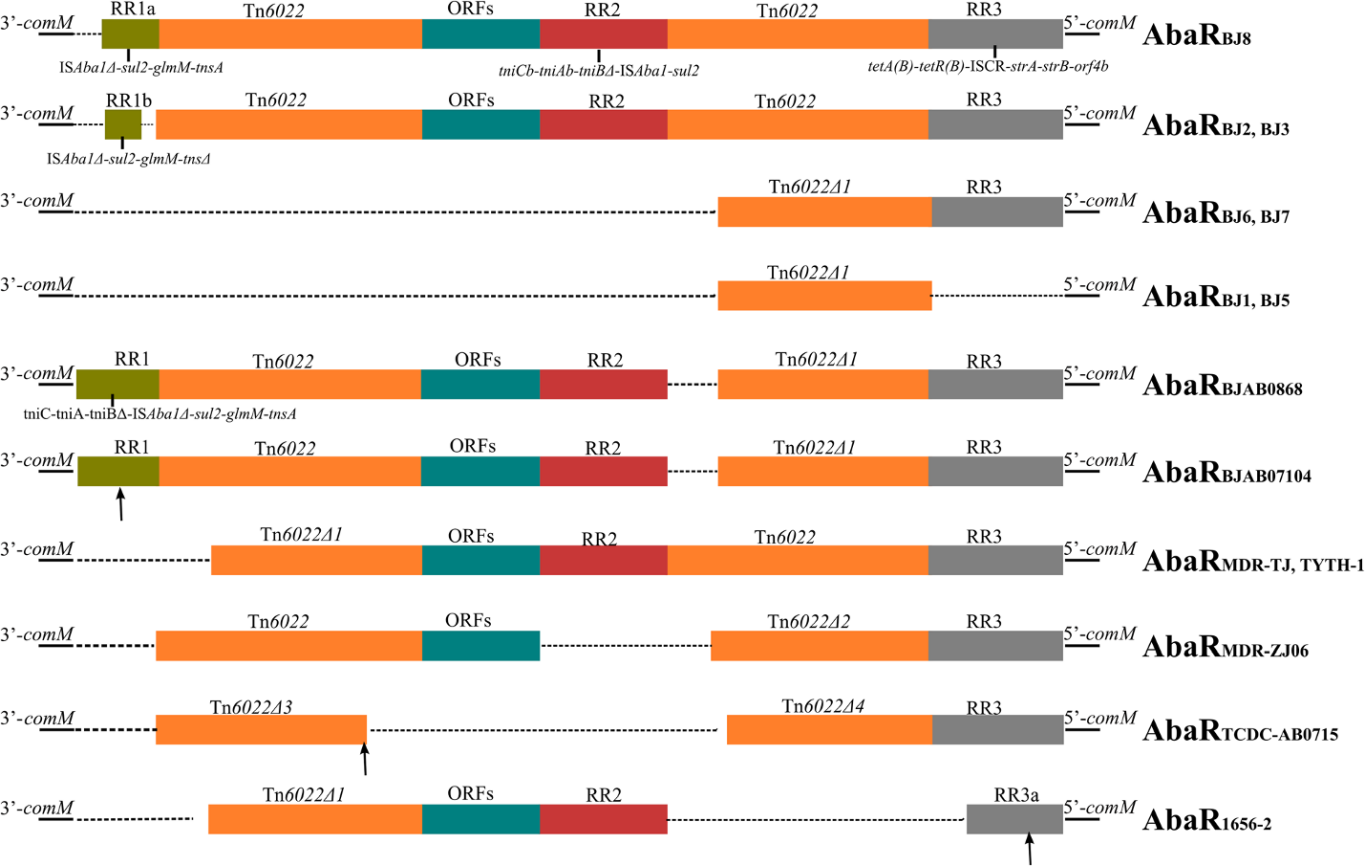
**Genetic structures of AbaR-like RIs in seven MDR strains and seven previously reported GC2 strains.** Green rectangles indicate resistance region RR1; red rectangles indicate resistance region RR2; grey rectangles indicate resistance region RR3; orange rectangles indicate Tn*6022* and Tn*6022Δ*; and blue rectangles indicate a cluster of *orfs* encoding proteins with unknown function. The dashed black lines denote deletions. The vertical arrows in AbaR_BJAB07104_, AbaR_1656–2_ and AbaR_TCDC-AB0715_ indicate insertions of segments specific to these strains. RR1: *tniC*, *tniA*, *tniBΔ*, IS*Aba1*, *sul2*, *glmM* (phosphoglucosamine mutase), and *tnsA* (transposase protein A); RR2: *tniCb*, *tniAb*, *tniBΔ2*, IS*Aba1*, *sul2*, and CRΔ; RR3: *tetA(B)*, *tetR(B)*, CR (IS*Vsa3*), *strB*, *strA*, and *orf4b.*

The region from 982 to 1,034 kb was predicted to be the prophage locus. The sequence of strain BJ1 in this locus is highly similar to that of the reference genome, while other strains have variable sequences in this region (low protein identity or missing). Interestingly, an IS*Aba1*-associated deletion of approximately 20 kb in a region of adhesion genes (*csuE*) from 1,343 to 1,363 kb was absent from the strain BJ2 and from the previously reported reference strain MDR-ZJ06 [23]. The region from 1,364 to 1,400 kb encompasses a cluster of genes involved in iron acquisition. The region from 1,575 to 1,617 kb was predicted to be the second prophage locus. The approximately 40-bp region from 2,460 to 2,500 kb, which encodes the entire type VI secretion system (T6SS), was completely absent from strain BJ4. The region from 3,672 to 3,710 kb, which encodes the entire AbaR-like genomic island (RIs), was also completely absent from strain BJ4. A more detailed analysis of this island is shown in Figure 4. The region from 3,798 to 3,894 kb contains many highly divergent genes, including several membrane proteins, stress-related proteins, and efflux pumps. Specifically, the region from 3,869 to 3,894 kb encompasses a series of genes encoding the O-antigen component of LPS.

In addition, the above-mentioned variable regions are always accompanied by several insertion elements, which may assist the integration of resistant and pathogenesis-related genes and facilitate the transfer of drug resistance and pathogenic genes among strains. In addition, IS elements may enhance drug-resistance and virulence by promoting gene expression [24, 25]. Furthermore, the CRISPR (clustered regularly interspaced short palindromic repeats) systems, which were identified in the genomes of three GC I strains (AYE, AB0057 and AB307-0294), were not present in any of the 8 sequenced strains.

### AbaR-like resistance islands (RIs)

We compared the sequences of AbaR-like RIs in each *A. baumannii* isolate and found a series of variation events at this locus (Figure 4). AbaR-like RIs inserted in the *comM* gene were identified in all 7 MDR strains: AbaR_BJ2_ and AbaR_BJ3_ shared the same structure, AbaR_BJ6_ and AbaR_BJ7_ shared a structure, and AbaR_BJ1_ and AbaR_BJ5_ shared a structure. In addition, AbaR_BJ8_, AbaR_BJ2_ and AbaR_BJ3_ were novel AbaR-like RIs, while the remaining regions have been previously reported [23, 26]. AbaR-like RIs were not found in strain BJ4, which may partly explain its susceptibility to antibiotics.

AbaR_BJ8_ shares the same backbone with AbaR_MDR-TJ_ and consists of two Tn*6022* transposons and 3 resistance-related regions (RR) (Figure 4). RR1a inserted at the 3’-end of the island contains IS*Aba1Δ* (mobile element), *sul2* (conferring sulfonamide resistance), *glmM* (phosphoglucosamine mutase) and *tnsA* (transposase protein A); RR2 located between the two copies of Tn*6022* bears the antibiotic resistance gene sul2; RR3 at the 5’ end of AbaR_BJ8_ contains four resistance genes: *tetA* and *tetR* (conferring tetracycline resistance), *strA* and *strB* (conferring streptomycin resistance). There was also a cluster of ORFs inserted between Tn*6022* and RR2; this cluster is designated “ORFs” in Figure 4. Compared with AbaR_BJ8_, the RR1a region and a part of the 3’end of Tn*6022* in AbaR_MDR-TJ_ were absent. AbaR_BJ2_ and AbaR_BJ3_ had a truncated RR1a (with a *tnsAΔ*) designated as RR1b; the rest of the structure was identical to that of AbaR_BJ8_. AbaR_BJ6_ and AbaR_BJ7_ included Tn*6022Δ1* and RR3 segments, while AbaR_BJ1_ and AbaR_BJ5_ only contained the Tn*6022Δ1* segment without resistance genes.

Comparative analysis of AbaR-like RIs in the 7 MDR strains and the other 7 GC II isolates was also performed (Figure 4). One major distinction among the RIs in the GC II strains was the presence or absence of a second Tn*6022* copy. Tn*6022* or Tn*6022Δ* were mutual segments that existed in all of these strains, while the three resistance regions (RR1, RR2 and RR3) and ORFs were deleted or truncated in certain strains. In AbaR_BJAB0714_, AbaR_TCDC-AB0715_ and AbaR_1656–2_, large fragments of inserted segments were found: the vertical arrow in AbaR_BJAB07104_ indicates the insertion of Tn*6206* and the *tra* system specific to this island [27]; the arrow in AbaR_1656–2_ indicates an inserted RR3a segment containing the resistance genes *per-1* and *strA* (*tnpA-tnpA2-gst-per-1-tnpA1-insB-IS3-strA*) but not the *tetA(B)* and *tetR(B)* regions of RR3 [28]; and the arrow in AbaR_TCDC-AB0715_ indicates a large segment specific to this strain containing six IS26 elements and multiple resistance genes [29].

**Figure 5:**
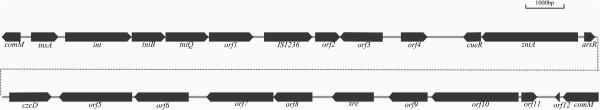
**Structure of genomic islands in the BJ4 strain.** The structure was drawn to scale according to the sequences of the genomic island in the genome and showed that the *comM* gene was interrupted by a novel genome island (designated as GI_BJ4_). This island was 29.3 kb in length and did not contain any antibiotic resistance genes.

Interestingly, the *comM* region of the drug-sensitive strain BJ4 was interrupted by a novel genome island (GI, designated GI_BJ4_). This island was 29.3 kb in length and contained no antibiotic resistance gene (Figure 5). Rather, five metal-resistance genes, including *cueR*, *zntA*, *arsR*, *czcD* and *xre* were identified within this island. Furthermore, 12 orfs of unknown function were present in GI_BJ4_, including a *tnsA* gene encoding an endonuclease domain protein, an *int* gene encoding an integrase core domain protein, the transposon-related *tniB* and *tniQ* genes and an inserted IS*1236* segment.

### Other mobile elements containing resistance genes

Class 1 integron is an important factor for the horizontal transfer of resistance genes in *A. baumannii*, especially the aminoglycoside resistance genes [30]. Six of our MDR strains contained a class 1 integron, and their gene cassette arrays were as follows: the integron of BJ1 harbored a gene cassette array of *aacC1*-*orfP*-*orfP*-*orfQ*-*aadA1*; the integrons of BJ2, BJ6 and BJ7 included gene a cassette array of *aacC1*-*orfA*-*orfB*-*aadA1*; and the integrons of BJ3 and BJ5 had a gene cassette array of *aacA4*-*catB8*-*aadA1*. Among these gene cassettes, *aacC1*, *aadA1* and *aacA4* are aminoglycoside resistance genes; *catB8* is group B chloramphenicol acetyltransferase; and *orf P*, *orfQ*, *orfA* and *orfB* encode proteins of unknown function. In addition, all of the found integrase protein sequences were 100% identical.

We also identified a *bla*_*OXA-23*_-containing transposon Tn*2009* in all sequenced strains, except for BJ4 and BJ8. This result was consistent with the antimicrobial susceptibility testing. Tn*2009* has been previously described in three Chinese *A. baumannii* strains: MDR-ZJ06, AB16 and MDR-TJ (in the pABTJ1plasmid) [22, 23, 30]. Genomic analysis revealed that Tn*2009* was flanked by two directed repeats of IS*Aba1* elements at both ends, and the class D β-lactamase gene *bla*_*OXA-23*_ existed at the 3’-end and was adjacent to the ISAba1 element. IS elements represent another source of variability among *A. baumannii* isolates, and the insertion of IS*Aba1* might play a significant role in the expression of *bla*_*OXA-23*_ [24, 25].

### Comparative analysis of antibiotic resistance genes

A comparative analysis of antibiotic resistance genes was performed on the 8 sequenced strains and 16 reference strains, among which the BJ4, D1279779, AB307-0294, ATCC17978 and SDF strains are antibiotic susceptible (Table 4). There are four types of β-lactamase in all of these strains, including class A β-lactamase, class C β-lactamase, class D β-lactamase and the metallo-β-lactamase superfamily. The four types of β-lactamase are encoded by various types of genes and are responsible for much of the multidrug resistance of these strains. Among the 8 sequenced strains in this study, the *ampC*, metallo-β-lactamase superfamily gene, putative class A β-lactamase gene and *bla*_*OXA-66*_ existed in all of the 7 MDR strains. The *tem-1* and putative class C β-lactamase gene were shared by six of the seven MDR strains. However, *per-1* is unique to BJ8, and *bla*_*OXA-69*_ is unique to BJ4. The *per-1* gene also exists in MDR strain 1656–2. *Per-1* is an extended-spectrum β-lactamase, and its induction might be responsible for resistance to all cephalosporins and cause difficulties in treating infections [31].

**Table 4:** **Antimicrobial resistance-associated genes in the 8 BJ strains and 16 reference strains. 0 = absent, 1 = present, 1R = present and resistant (bearing a point mutation), 1S = present but sensitive (no point mutation)**

Drug class	Enzyme class, description	Coding gene	BJ1	BJ2	BJ3	BJ4	BJ5	BJ6	BJ7	BJ8	AYE	ACICU	TYTH-1	AB0715	1656-2	ZW85-1	D1279779	AB0057	AB307-0294	ATCC17978	MDR-TJ	MDR-ZJ06	BJAB07104	BJAB0868	BJAB0715	SDF
β-Lactamases	class A β-lactamase	*tem-1*	1	1	0	0	1	1	1	1	0	0	0	1	0	0	0	1	0	0	0	0	0	1	0	0
		*per-1*	0	0	0	0	0	0	0	1	0	0	0	0	1	0	0	0	0	0	0	0	0	0	0	0
		*veb*	0	0	0	0	0	0	0	0	1	0	0	0	0	0	0	0	0	0	0	0	0	0	0	0
	putative class A β-lactamase		1	1	1	0	1	1	1	1	0	1	1	1	1	0	0	1	0	1	1	1	1	1	1	0
	class C β-lactamase	*ampC*	1	1	1	1	1	2	1	1	1	3	1	2	2	0	1	2	1	1	4	2	3	3	2	0
	putative class C β-lactamase		1	1	1	1	1	0	1	1	0	0	1	0	0	0	0	0	0	0	0	0	0	0	1	0
	class D β-lactamase	*bla*_*OXA-23*_	1	1	1	0	1	1	1	0	0	0	0	1	0	0	0	1	0	0	1	1	1	1	1	0
		*bla*_*OXA-66*_	1	1	1	0	1	1	1	1	0	1	0	1	0	0	0	0	0	0	1	1	1	1	0	0
		*bla*_*OXA-69*_	0	0	0	1	0	0	0	0	1	0	0	0	0	0	0	1	1	0	0	0	0	0	0	0
		*bla*_*OXA-10*_	0	0	0	0	0	0	0	0	1	0	0	0	0	0	0	0	1	0	0	0	0	0	0	0
		*bla*_*OXA-90*_	0	0	0	0	0	0	0	0	0	0	0	0	0	1	0	0	0	0	0	0	0	0	0	0
		*bla*_*OXA-109*_	0	0	0	0	0	0	0	0	0	0	0	0	1	0	0	0	0	0	0	0	0	0	0	0
	metallo-β-lactamase superfamily		2	2	1	1	2	2	2	2	1	1	0	2	0	4	5	5	7	1	2	1	1	1	0	0
Aminoglycosides	aminoglycoside N-acetyltransferase	*aacA4*	0	0	1	0	1	0	0	0	0	1	1	1	0	0	0	0	0	0	1	1	1	1	0	0
		*aacC1*	1	1	0	0	0	1	1	0	1	0	0	1	1	0	0	1	0	0	1	1	0	0	0	0
		*aac3iia*	0	0	0	0	0	0	0	0	0	0	0	0	0	0	0	0	0	0	0	0	0	0	1	0
	aminoglycoside O-phosphotransferase	*strA*	0	1	1	0	0	1	1	1	1	0	1	1	2	0	0	0	0	0	1	1	1	1	1	0
		*strB*	0	1	1	0	0	1	1	1	1	0	1	1	1	0	0	0	0	0	1	1	1	1	1	0
		*aphA1*	0	1	1	1	0	1	1	1	2	1	1	3	1	1	1	2	1	0	1	2	2	2	0	1
		*aph3via*	0	0	0	0	0	0	0	0	0	0	0	0	0	1	0	0	0	0	0	0	0	0	1	0
	aminoglycoside O-adenylyltransferase	*aadA1*	1	1	1	0	1	1	1	0	2	0	1	2	2	0	0	1	0	0	2	2	1	1	0	0
		*ant2ia*	0	0	0	0	0	0	0	0	1	0	0	0	0	0	0	0	0	0	0	0	0	0	0	0
Chloramphenicol	group A chloramphenicol acetyltransferase	*catA1*	0	0	0	0	0	0	0	0	1	0	0	0	0	0	0	1	0	0	0	0	0	0	0	0
	group B chloramphenicol acetyltransferase	*catB8*	0	0	1	0	1	0	0	0	0	0	0	0	0	0	0	0	0	0	0	1	0	0	0	0
		*catB3*	0	0	0	0	0	0	0	0	0	0	1	1	0	0	0	0	0	0	1	1	1	1	0	0
Sulfonamides	sulfonamide-resistant dihydropteroate synthase	*sul1*	1	1	1	0	1	1	1	1	3	1	1	2	1	0	0	2	0	0	2	2	1	1	0	0
		*sul2*	0	1	1	0	0	0	0	1	0	0	1	1	1	1	0	0	0	1	1	0	1	1	1	0
Fluoroquinolones	gyrA mutation (Ser-83 → Leu)	*gyrA*	1R	1R	1R	1S	1R	1R	1R	1R	1R	1R	1R	1R	1R	1S	1S	1R	1R	1S	1R	1R	1R	1R	1R	1S
	parC mutation (Ser-80 → Ile)	*parC*	1R	1R	1R	1S	1R	1R	1R	1S	1R	1S	1S	1R	1S	1S	1S	1R	1S	1S	1S	1S	1S	1S	1S	1S
Efflux pumps	MFS (major facilitator superfamily) family	*tetA*	0	1	1	0	0	1	1	0	1	0	0	0	0	0	0	1	0	0	0	0	0	0	0	0
		*tetB*	0	0	0	0	0	0	0	0	0	0	1	1	0	1	0	0	0	0	1	1	1	1	1	0
		*tetG*	0	0	0	0	0	0	0	0	1	0	0	0	0	0	0	0	0	0	0	0	0	0	0	0
		*cmlA*	0	0	0	0	0	0	0	0	1	0	0	0	0	0	0	0	0	0	0	0	0	0	0	0
		*cmlA5*	0	0	0	0	0	0	0	0	1	0	0	0	0	0	0	0	0	0	0	0	0	0	0	0
	RND (resistance-nodulation-division) family	*adeA*	1	1	1	1	1	1	1	1	1	1	1	1	1	1	1	1	1	2	1	1	1	1	0	0
		*adeB*	1	1	1	1	1	1	1	1	1	1	1	1	1	1	1	1	1	1	1	1	1	1	0	0
		*adeC*	1	1	1	1	1	1	1	1	1	1	1	1	1	0	0	1	1	0	1	1	1	1	0	0

Resistance to aminoglycosides is primarily mediated by aminoglycoside-modifying enzymes (AMEs), which include three types: aminoglycoside N-acetyltransferase, aminoglycoside O-phosphotransferase, and aminoglycoside O-adenylyltransferase. These three types of AME genes, especially the *aacA4*, *aacC1*, *strA*, *strB*, *aphA1* and *aadA1* genes, are commonly found in the 7 sequenced MDR BJ strains and the 12 reference MDR strains. In contrast, the sequenced non-MDR strain BJ4 and the 4 reference non-MDR strains contain fewer AMEs genes: BJ4, D1279779, AB307-0294 and SDF each contain one aphA1gene, and ATCC17978 does not contain an AME gene. Mutations in the *gyrA* (Ser83Leu) and *parC* (Ser80Ile) genes are responsible for quinolone resistance in *A. baumannii*. In this analysis, all of the MDR strains except ZW85-1 contained a mutation in *gyrA* gene, while the *parC* gene mutation was only present in nine of the MDR strains. This result might indicate that *gyrA* (Ser83Leu) plays a more important role than does *parC* (Ser80Ile) in fluoroquinolone resistance. Among the 19 MDR strains, only 10 include the group A or group B chloramphenicol acetyltransferase genes. There may be other resistance mechanisms, such as efflux pumps, that contribute to chloramphenicol resistance in these MDR strains. The sulfonamide-resistant dihydropteroate synthase genes were present in all of the analyzed MDR strains, while in the 5 non-MDR strains, only ATCC17978 contained a *sul2* gene.

The RND (resistance-nodulation-division) family efflux pump, consisting of the *adeA*, *adeB* and *adeC* genes, was present in most of these strains. This efflux pump requires the coexistence of all three genes (*adeA*, *adeB* and *adeC*) to function properly. The antibiotic-susceptible strains D1279779, ATCC17978 and SDF do not contain a functional AdeABC efflux pump. All of the MDR strains except BJAB0715 contain intact *adeA*, *adeB* and *adeC* genes, which might play a role in their antibiotic resistance. Some efflux pump genes belonging to the MFS (major facilitator superfamily) were also identified in several of the MDR strains, including tetA, tetB and tetG, which encode tetracycline efflux proteins, and cmlA and cmlA5, which encode chloramphenicol efflux proteins.

## Discussion

In this study, we used whole-genome sequencing methods to characterize genomic variations and antibiotic resistance mechanisms in clinical *A. baumannii* isolates with various sequence types and drug susceptibility profiles. Although the isolates are closely related, we identified significant genetic differences and a high degree of genomic plasticity in these strains. Pan-genomic analysis of the 8 *A. baumannii* isolates and the other 16 complete genomes revealed that *A. baumannii* genomes were highly heterogeneous with respect to gene content and possessed a series of unique genes; these results are similar to those of previous studies [15, 32]. The unique genes are enriched in COG class L (Replication, recombination and repair), class V (Defense mechanisms), and class M (Cell wall/membrane/envelope biogenesis), which suggests that these genes are critical for *A. baumannii* survival or are closely associated with the ability of the bacteria to adapt to challenging niches.

Phylogenetic analysis showed that the drug-susceptible isolate BJ4 was distinct from the other MDR strains, and its closer relationship with the AB0057 and AYE MDR isolates offers another perspective on the origins and acquisition of antibiotic resistance determinants. In addition, the close relationship among strains BJ2, BJ6, and BJ7 indicated they these strains may come from a common ancestor. The *csuE* deletion in strain BJ2 suggested that this loss may have occurred after the ancestral strain entered the hospital, followed by the mixing of strains with and without *csuE*.

A comparison of the gene content-based dendrogram with the core SNP tree revealed a similar clustering relationship. The slight difference in tree topology is primarily driven by (i) lateral gene transfer and (ii) IS-mediated phage- and plasmid-associated gene gain and loss. The CRISPR repeat elements, which are involved in a complex mechanism that inhibits invasive phage and plasmid DNA, were not present in any of the eight strains, which may partly explain the widespread distribution of phage- and plasmid-related genes and the extensive genomic plasticity among *A. baumannii* isolates.

Many regions associated with IS-mediated deletions, including a deletion of the entire T6SS region, have been shown to be involved in interbacterial interactions [12]. We found that the T6SS region is conserved in all 7 of the analyzed MDR *A. baumannii* isolates. As antibiotic therapy appears to reduce interbacterial competition, this result is consistent with the hypothesis that the MDR phenotype is conferred by antibiotic resistance genes, indicating that the T6SS regions are less important [33]. The surface polysaccharide loci are highly variable among the 8 strains, which is consistent with a previous report that these regions are significant sources of variability within *A. baumannii* strains [14]. We also found that the OC locus (from 558 to 566 kb in Figure 3) was less variable and was highly conserved among the MDR strains, but this region was almost completely missing from the drug-sensitive strain BJ4. In addition, virulence gene analysis showed that a total of 10 putative virulence genes were present in all *A. baumannii* genomes, suggesting that these genes may play significant roles in the pathogenesis of *A. baumannii* infection.

AbaR-like RIs inserted in the *comM* gene were identified in all 7 of the analyzed MDR strains but not in the drug-sensitive strain BJ4. This isolate contained a 29.3-kb new GI with five metal-resistance genes but no antibiotic resistance genes (Figure 5). Therefore, we suggested that GIs inserted into the *comM* gene are not always associated with antibiotic resistance, and their function might be related to the adaption of the strain to its survival niche. We also found that the RI is highly variable in composition and is not the only contributor to the MDR phenotype. Resistance genes in other mobile elements are found outside the RIs, and they are able to contribute to drug resistance in each strain examined. Among the seven MDR strains, only strain BJ8 did not contain the *bla*_*OXA-23*_-containing transposon Tn*2009*, suggesting that Tn2009 is an important carrier of the *bla*_*OXA-23*_ gene in clinical isolates. Furthermore, the detection of Tn2009 in both chromosome and plasmid DNA suggested that this transposon can be transferred easily between clinical strains.

Antimicrobial susceptibility testing indicated that, compared with the 7 MDR strains, the drug-sensitive strain BJ4 shows low-level resistance to piperacillin-tazobactam (TZP), ceftriaxone (CTR) and ceftazidime (CAZ). We hypothesize that this type of low-level resistance is likely caused by the four β-lactamase genes carried by this strain (Table 4). In addition, the *aphA1* gene identified in BJ4 encodes resistance to kanamycin but not to gentamicin, amikacin or netilmicin [34]. This result may explain why this strain is susceptible to gentamicin and amikacin. The class D β-lactamase gene *bla*_*OXA-69*_ is unique to strain BJ4, while the 7 MDR strains contain a *bla*_*OXA-66*_ gene at the same genomic location. The intrinsic *bla*_*OXA-69*_ gene encodes oxacillinase, which can hydrolyze imipenem and meropenem at a low level [35]. It is reported that the presence of IS elements such as ISAba1 in the upstream of *bla*_*OXA-69*_ can up-regulate the resistance gene’s expression level [25, 36]; however, few IS elements were found in the drug-sensitive strain BJ4, and no IS elements are present upstream of the intrinsic resistance genes. In contrast, the MDR strains contain a series of IS element copies, including ISAba1, ISAba125, and IS26; importantly, ISAba1 elements were identified upstream of *bla*_*OXA-23*_ and other RIs in all MDR strains. These results may partly explain the variations in antimicrobial susceptibility between the MDR strains and the sensitive strain.

SNPs are another important source of genetic variation and may contribute to drug resistance and pathogenesis in *A. baumannii* [37]. Both phylogenic and SNP analysis indicated that the drug-sensitive isolate BJ04 is genetically distinct from other MDR *A. baumannii* strains. In addition, only a small proportion of SNPs are nonsynonymous among closely related clinical MDR *A. baumannii* strains with the same STs, indicating that these strains may undergo purifying selection on a genome-wide scale. Furthermore, mutation hotspots between MDR strains were identified in several genes associated with drug resistance, e.g., genes encoding dihydropteroate synthase, a target for sulfonamide antibiotics, and the putative RND superfamily exporter genes, which encode multidrug efflux pumps [38].

## Conclusions

In this study, we used whole-genome sequencing to identify genetic variants in *A. baumannii* isolates. We performed comparative genomic analysis of 8 clinical *A. baumannii* isolates with 16 available complete *A. baumannii* genomes in the NCBI database. Our results shed new light on the importance of genomic variations, especially transposon-related and/or phage-related gene variations, in the evolution of *A. baumannii*. Furthermore, we suggest that the MDR *A. baumannii* strains harbor diverse antibiotic resistance mechanisms. Future studies focused on a larger sample of *A. baumannii* isolates from various hospitals and lineages are necessary to better understand the rapid development of antibiotic resistance in *A. baumannii*.

## Methods

### Bacterial isolates and antimicrobial susceptibility testing

*The A. baumannii* strains BJ1 to BJ8 were isolated from the 306^th^ Hospital of People’s Liberation Army in Beijing, China. Identification of the isolates and antimicrobial susceptibility testing were performed using the bioMérieux VITEK-2 AST-GN13 system following the manufacturer’s instructions. The minimum inhibitory concentration (MIC) of 11 antimicrobial agents was determined according to the recommendations given by the Clinical and Laboratory Standards Institute (CLSI) (Clinical and Laboratory Standards Institute, 2011) [1]. The reference strains *Escherichia coli* ATCC 25922 and *Pseudomonas aeruginosa* ATCC 27853 were used as quality controls.

### DNA extraction, whole-genome sequencing, and annotation

Genomic DNA was extracted using the TIANamp bacteria DNA kit (Tiangen Biotech (Beijing) Co., Ltd.) according to the manufacturer’s instructions. The genomic DNA was fragmented by ultrasonication, and the DNA fragments were subjected to the whole-genome sequencing workflow of the Illumina HiSeq 2000 system. Genome assembly was carried out by SOAPdenovo (http://soap.genomics.org.cn). The detailed methods for genome assembly and annotation were described in another study [39]. To close gaps within the AbaR-like RIs and integrons, primer pairs were designed at the end of each gap using the genomes as templates. The PCR products were sequenced with an ABI 3730 automated DNA sequencer, and the sequences of these products were used to fill the gaps. Random primers within the AbaR-like RIs and integron regions were subsequently designed to reconfirm the accuracy of these sequences.

### Multiple locus sequence typing (MLST)

To identify sequence types, we aligned the assembled sequences against seven housekeeping gene sequences (*gltA*, *gyrB*, *gdhB*, *recA*, *cpn60*, *gpi*, and *rpoD*) using BLAST and then extracted the aligned sequences by comparing them to allele profiles in the *A. baumannii* MLST database (http://pubmlst.org/).

### SNP detection and analysis

The short reads were first aligned onto the MDR-TJ genome reference using the SOAP2 program [40]. Then, SOAPsnp was used to score SNPs from aligned reads [41]. The SOAPsnp results were filtered as follows: 1) the read coverage of the SNP site was greater than five; 2) the Illumina quality score of either allele was greater than 30; and 3) the count of the all of the mapped best base was more than twice the count of all of the mapped second best base. From all of the SNPs identified in the sequenced genome sequences, the SNP density was calculated throughout the MDR-TJ genome using a sliding-window size of 5 kb. This window was moved at steps of 1 kb at a time, and the SNP number within each window size was counted. The construction of an SNP clustering map was performed using Circos [42].

### Comparative genomics analysis

Genomic data used in comparative analysis were downloaded from the NCBI ftp server, including complete genome sequences of *A. baumannii* isolates MDR-ZJ06 (CP001937.1), MDR-TJ (CP003500.1), BJAB0715 (CP003847.1), AB1656-2 (CP001921.1), AB0057 (CP001182.1), AB307-0294 (CP001172.1), ACICU (CP000863.1), ATCC 17978 (CP000521.1), AYE (CU459141.1), BJAB07104 (CP003846.1), BJAB0868 (CP003849.1), D1279779 (CP003967.1), TCDC-AB0715 (CP002522.2), TYTH-1 (CP003856.1), ZW85-1 (CP006768.1), SDF (CU468230), and ADP1 (NC_005966.1).

Multiple sequence alignments of the *A. baumannii* genomes were performed with Mugsy [43]. The phylogenetic tree was constructed using dnaml from the PHYLIP package [44] based on SNPs from the whole-genome alignment, and the genome of Acinetobacter sp. ADP1 was used as the outgroup. An all-against-all BLASTP search between every pair of protein sequences from each strain was performed. Orthologs were identified using PanOCT [17] with the BLASTP output (Identity 80%; Aligned length 30%; E-value < 1e^−5^). The map for core and pan-genome calculations in *A. baumannii* isolates was performed using PanGP [45]. The heatmap figure was generated using the *R* package pheatmap [46]. The map of ORF comparisons among *A. baumannii* genomes was constructed using Circos [42]. As shown in the phylogenetic analysis, the strain SDF was genetically the most distant from our strains; this strain is therefore more suitable for analyzing the nonsynonymous substitution rate and thereby defining the rapidly and slowly evolving protein-coding genes. We aligned the amino acid sequences of SDF with our sequenced strains, estimating the nonsynonymous substitution rates of orthologs based on the NG method using KaKs_Calculator 2.0 [47].

COG annotation was performed using the BLAST software against the COG database. COG enrichment analysis was determined using Fisher’s exact test by comparing the prevalence of a target group of genes assigned to a specific COG category to the prevalence of genes in the whole genome assigned to that COG category. To identify possible virulence factors, the Virulence Factors Database (VFDB) [48] was aligned to the ORF protein sequences and filtered with 60% identity and 90% aligned length. To search the antibiotic resistance genes, the protein-coding sequences were aligned against the Antibiotic Resistance Database (ARDB) [49, 50] using the similarity thresholds recommended in ARDB. PHAST [51] was used to identify the putative prophages in Acinetobacter genomes. ISs were identified using the IS Finder database (http://www-is.biotoul.fr) [52]. The detection of CRISPR loci in 8 sequenced draft genome sequences was performed using CRISPRFinder [42].

### Nucleotide Sequence Accession Numbers

The genome sequences of *A. baumannii* strains from BJ1 to BJ8 reported in this study have been deposited in GenBank under accession numbers JPLF00000000, JPLG00000000, JPLH00000000, JPLI00000000, JPLJ00000000, JPLK00000000, JPLL00000000, and JPLM00000000, respectively.

## Electronic supplementary material

Additional file 1: Table S1: Orthologous groups and unique CDS. Detailed information for the *A. baumannii* pan-genome analysis used in this study. “1” and “0” indicate that the gene is present and absent, respectively, from the individual strain. (XLS 2 MB)

Additional file 2: Table S2: Comparison of pair-wise SNP numbers between various strains using MDR-TJ as a reference. (XLS 20 KB)

Additional file 3: Figure S1: SNP density map constructed using Circos. The red bars indicate regions with significantly high SNP density. The scale bar within the circle indicates the number of SNPs. (PNG 1 MB)

Additional file 4: Table S3: Putative virulence genes identified in the 8 sequenced strains and 16 reference strains using VFDB. “1” and “0” indicate that the gene is present and absent, respectively, from the individual strain. (XLS 29 KB)
