# Supplementary figures and images for: Comparative genomic analysis of Acinetobacter baumannii clinical isolates reveals extensive genomic variation and diverse antibiotic resistance determinants
Source: BMC Genomics. 2014 Dec 22;15(1):1163. doi: 10.1186/1471-2164-15-1163 (PMC4367897; doi:10.1186/1471-2164-15-1163)

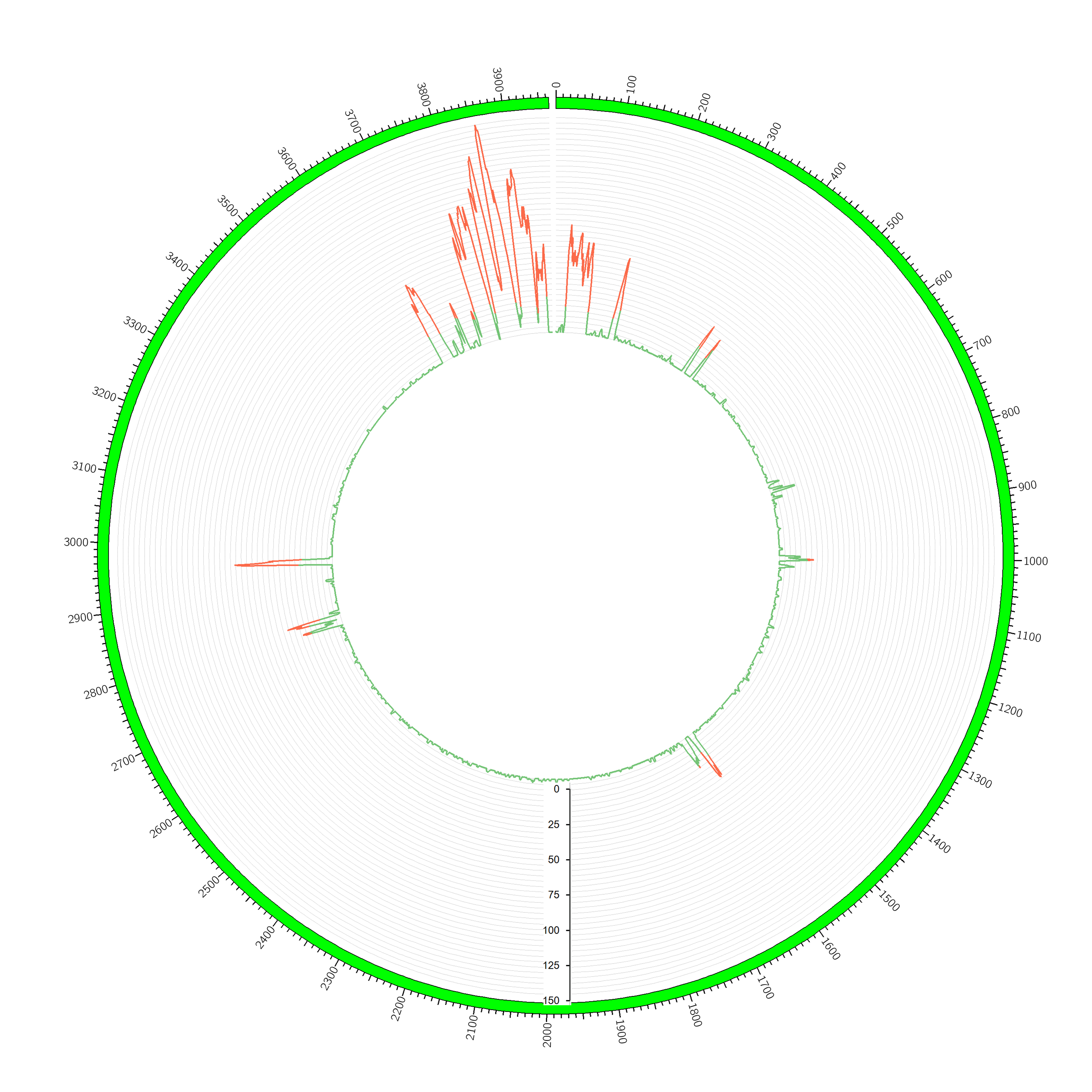

Supplement: Supplementary file 3 — Additional file 3: Figure S1: SNP density map constructed using Circos. The red bars indicate regions with significantly high SNP density. The scale bar within the circle indicates the number of SNPs. (PNG 1 MB) [file 12864_2014_6893_MOESM3_ESM.png]
